# A step forward to the optimized HlyA type 1 secretion system through directed evolution

**DOI:** 10.1007/s00253-023-12653-7

**Published:** 2023-07-05

**Authors:** Zohreh N. Pourhassan, Haiyang Cui, Neele Muckhoff, Mehdi D. Davari, Sander H. J. Smits, Ulrich Schwaneberg, Lutz Schmitt

**Affiliations:** 1 Institute of Biochemistry, Heinrich Heine University, Universitätsstr. 1, 40225 Düsseldorf, Germany; 2 Institute of Biotechnology, RWTH Aachen University, Worringerweg 3, 52074 Aachen, Germany; 3 DWI-Leibniz Institute for Interactive Materials, Forckenbeckstraße 50, 52056 Aachen, Germany; 4 Present Address: Carl R. Woese Institute for Genomic Biology, University of Illinois at Urbana-Champaign, 1206 West Gregory Drive, Urbana, IL 61801 USA; 5 Department of Bioorganic Chemistry, Leibniz Institute of Plant Biochemistry, Weinberg 3, 06120 Halle, Germany

**Keywords:** Protein secretion, KnowVolution, Gram-negative bacteria, Protein engineering, Directed evolution, Bacterial secretion system

## Abstract

**Abstract:**

Secretion of proteins into the extracellular space has great advantages for the production of recombinant proteins. Type 1 secretion systems (T1SS) are attractive candidates to be optimized for biotechnological applications, as they have a relatively simple architecture compared to other classes of secretion systems. A paradigm of T1SS is the hemolysin A type 1 secretion system (HlyA T1SS) from *Escherichia coli* harboring only three membrane proteins, which makes the plasmid-based expression of the system easy. Although for decades the HlyA T1SS has been successfully applied for secretion of a long list of heterologous proteins from different origins as well as peptides, but its utility at commercial scales is still limited mainly due to low secretion titers of the system. To address this drawback, we engineered the inner membrane complex of the system, consisting of HlyB and HlyD proteins, following KnowVolution strategy. The applied KnowVolution campaign in this study provided a novel HlyB variant containing four substitutions (T36L/F216W/S290C/V421I) with up to 2.5-fold improved secretion for two hydrolases, a lipase and a cutinase.

**Key points:**

*• An improvement in protein secretion via the use of T1SS*

*• Reaching almost 400 mg/L of soluble lipase into the supernatant*

*• A step forward to making E. coli cells more competitive for applying as a secretion host*

**Graphical Abstract:**

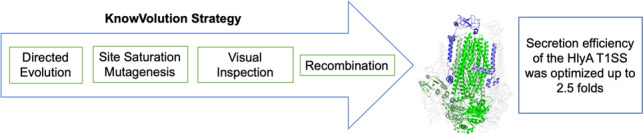

**Supplementary Information:**

The online version contains supplementary material available at 10.1007/s00253-023-12653-7.

## Introduction

Secretion of proteins is important for recombinant protein production as it can lead to protein production in high quality and yield, while at same time reducing the production costs (Kleiner-Grote et al. 2018). Protein secretion in Gram-negative bacteria, such as *Escherichia coli*, often results in low titers. This limitation is of particular concern and reduces the general utilities of biotechnological hosts, such as *E. coli*, as secreting hosts (Burdette et al. 2018; Kleiner-Grote et al. 2018).

To enhance the secretion efficiency of different classes of secretion systems, different approaches have been applied. For instance, these approaches include modification of secretion signal (Freudl 2018), transferring the secretion system to a more efficient secretion host (Eom et al. 2014), and modification of genetical elements such as enhancer fragment (Pourhassan et al. 2022a). In addition to these approaches, protein engineering has proved to be a powerful tool for tailoring protein properties (Wong et al. 2006). Until now, different secretion systems have been subjected to various engineering approaches, but these efforts have commonly had little success due to the complexity of the secretion systems (Burdette et al. 2018).

Among different secretion systems of Gram-negative bacteria, type 1 secretion systems (T1SS) have relatively simple architecture, and this makes them attractive targets to be optimized (Burdette et al. 2018). T1SS translocates proteins in an unfolded state across both membranes of Gram-negative bacteria in a single step. Subsequently, proteins fold in their functional state in the extracellular space (Holland et al. 2016).

The T1SS paradigm is the hemolysin A type 1 secretion system (HlyA T1SS). The HlyA T1SS machinery consists of three membrane proteins: the ABC transporter HlyB, the membrane fusion protein HlyD, and the outer membrane protein TolC. TolC is recruited upon an interaction between the substrate (HlyA) and the inner-membrane complex (HlyB and HlyD). Then, these membrane proteins form a tunnel-channel redirecting HlyA from the cytosol to the extracellular space (Holland et al. 2016; Pourhassan et al. 2021).

Recently, the single particle cryo-EM structure of HlyB/HlyD complex revealed that a trimer of HlyB dimers, each associated with two molecules of HlyD (per HlyB dimer), forms the inner membrane of the system complex (Zhao et al. 2022).

HlyB is an ATP-binding cassette (ABC) transporter that powers the secretion of HlyA by hydrolyzing ATP. HlyB has a domain organization consists of a transmembrane domain (TMD) composed of six transmembrane helices (TMH), a hydrophilic cytoplasmic nucleotide-binding domain (NBD), and an N-terminal cytosolic domain called the C39-peptidase like domain (CLD) (Lecher et al. 2012; Schmitt et al. 2003). In addition to its ATPase activity, NBD has a supplementary function. Specifically, it interacts with HlyA prior to secretion, which is proposed to represent the substrate recognition step (Benabdelhak et al. 2003; Pourhassan et al. 2022b). Based on a series of pull-down assays, Lecher et al. suggested that the CLD is involved in positioning HlyA into the secretion pathway, indicating a chaperone-like activity for this domain (Lecher et al. 2012).

HlyD, also refers as the periplasmic adaptor protein of the system, interacts with HlyB and TolC to stabilize and seal the tunnel-channel of HlyA T1SS (Alav et al. 2021; Holland et al. 2016). In terms of topology, HlyD has an N-terminal cytosolic domain (60 residues) that follows by a single transmembrane helix (20 residues), and a large periplasmic region (Schtilein et al. 1992). A crystal structure of a soluble fragment of HlyD (lacking some of both N-terminal and C-terminal residues) became available. Based on this structural information, a hexameric state of HlyD was modeled (Kim et al. 2016).

Within various protein engineering strategies, KnowVolution is a general applicable protein engineering strategy to improve protein properties (Cheng et al. 2015). KnowVolution, also refers as knowledge gaining directed evolution, combines directed evolution and computational analysis in order to maximize improvements with a minimal experimental effort. A KnowVolution campaign is divided into four phases: (I) identification; (II) determination; (III) computational analysis; (IV) recombination (Yang et al. 2015). KnowVolution has been reported for the successful engineering of, e.g., the ligase PigC (Brands et al. 2021), laccases (Novoa et al. 2019), aryl sulfotransferases (Islam et al. 2018), and polymer-binding peptides (Rübsam et al. 2018).

HlyA1 or HlyAc, a 23-kDa C-terminal fragment of HlyA containing the secretion signal plus three GG repeats, is a carrier for the secretion of heterologous proteins (Nicaud et al. 1986). While it has been successful in secreting various proteins, its potential as a universal secretion platform is limited by low secretion titers. Improving the efficiency of HlyA T1SS for recombinant proteins remains a challenge (Pourhassan et al. 2021).

To optimize the secretion efficiency of the system, we recently worked on the HlyA enhancer fragment and have achieved great success (Pourhassan et al. 2022a), which promoted us to a further improvement of the system. In the current study, we report a two-round based KnowVolution campaigns on the inner membrane complex of HlyA T1SS (HlyB and HlyD) to achieve further improved HlyA T1SS secretion. The KnowVolution on HlyB yielded a variant with four beneficial substitutions that increased the secretion efficiency of lipase for 2.5-folds. In addition, the general applicability of the approach was also investigated with a cutinase as a second model system.

## Materials and methods

### Materials

All chemicals used in this study were purchased from Sigma-Aldrich, Roche Diagnostics GmbH, and Applichem GmbH if not stated otherwise. Enzymes were purchased from New England Biolabs (England), Promega GmbH (Germany), and SeSam-Biotech GmbH (Germany). All oligonucleotides were purchased from either Eurofins MWG Operon or GeneScript. The commercial available kits NucleoSpin plasmid miniprep kit and PCR clean-up kit were purchased from Macherey Nagel (Düren, Germany). Gibson assembly master mix was purchased from New England Biolabs (England). DNA sequencing was performed by Microsynth Seqlab (Göttingen, Germany).

### Generation of hlyB and hlyD casting error-prone PCR mutant library

For cloning and library generation, *E. coli* BL21-Gold (DE3) was used. Oligonucleotides used in this work are listed in Tables S1 and S2. Plasmid pK184_*EF*(Best)-*lipA*-*hlyA1*-*Ter*-*hlyBD* (Pourhassan et al. 2022a) was used as the backbone vector for the library generation. Note that the gene encoding the TolC protein is endogenous, and the genes encoding HlyB and HlyD are plasmid-based. The abbreviations in the name of the plasmid stand for: *EF* for enhancer fragment, *lip*A for *lipase* gene from *Serratia marcescens*, *hly* for hemolysin, and *Ter* for a terminator region. The *lac* promoter of plasmid pK184 is inducible with isopropyl β-D-1-thiogalactopyranoside (IPTG). This plasmid contains a kanamycin resistance gene.

Casting error-prone PCR (cepPCR) was used for generation of random mutagenesis libraries for HlyB and HlyD according to the published cepPCR protocol (Yang et al. 2017). Accordingly, the *hlyB* gene from *E. coli* UTI89, except of the region encoding for the NBD, was divided into six fragments, listed in Table S1.

The *hlyD* gene from *E. coli* UTI89 was divided into six fragments, listed in Table S1. For each fragment, a primer set, forward and reverse, was designed and employed for amplification of the fragment via epPCR.

The parameters and reaction conditions of cepPCR (50 µL) were as follows: 1X ThermoPol buffer, 0.2 mM dNTP mix, 400 pM of each primer, 10 U polymutarase polymerase, 60 ng DNA template, 0.1–0.9 mM MnCl_2_. The PCR was performed as follows: 94 °C for 2 min (1 cycle), 94 °C for 30 s, primer melting temperature (Tm) for 30 s, 68 °C for 1 min (for 30 cycles), and 68 °C for 10 min (1 cycle). To obtain mutations of the first amino acids of each fragment, the forward primers annealed upstream of reverse primer of the previous fragment. PCR products were checked on agarose gel and purified with the PCR clean-up kit according to the instructions of the manufacturer.

For cloning of the individual mutant fragments into plasmid pK184_*EF*(Best)-*lipA*-*hlyA1*-*Ter*-*hlyBD*, either the megawhop PCR or Gibson assembly was used, with the following details.

For each individual fragment, nine mutant libraries applying different MnCl_2_ concentrations (0.1 to 0.9 mM) were generated. The inactive ratio of libraries were determined by prescreening on tributyrin agar plates (trypton 10 g/L, yeast extract 5 g/L, NaCl 10 g/L, agar 15 g/L, gum arabic 1.5 g/L, tributyrin 15 g/L, kanamycin 50 μg/mL). The condition of library generation for each fragment was adjusted to obtain libraries with an inactive ratio of 40–50%. To construct the entire vector, the amplified fragment containing mutations was used as either megaprimer for megawhop PCR or insert for Gibson assembly.

The megawhop PCR was performed based on the published protocol (Miyazaki 2011). The megawhop PCR products were digested with DpnI (20 U, 37 °C, overnight) and purified using the PCR clean-up kit.

For Gibson assembly cloning, the vector template lacking the related fragment was amplified using a high-fidelity polymerase, Q5 polymerase. The PCR mixture (50 μL) contained 50 ng of template plasmid, 1 × Q5 standard reaction buffer, 0.2 mM dNTP mix, 200 pM of each primer, and 1 U Q5 DNA polymerase. Subsequently, the epPCR product of the related fragments and the PCR product of the backbone vector were digested with DpnI enzyme (overnight, at 37 °C) to remove any undigested wildtype vector. The PCR products were purified using a PCR clean-up kit. The whole plasmid construction of the epPCR product and the linear plasmid was performed via Gibson assembly according to the instructions of the manufacturer.

Subsequently, 2 μL of the assembled product of Gibson assembly reaction or the megawhop PCR product was transformed into chemically competent cells of *E. coli* BL21-Gold (DE3). The transformed cells were cultured directly on tributyrin LB agar plates containing kanamycin 50 μg/mL. Ten single cell clones of each library were picked and sent for sequencing. For each fragment, single active clones were picked up and cultured in 96-well plates (PS-F-bottom, Sarstedt, Germany).

### Site-saturation mutation of potential beneficial positions

Site-saturation mutagenesis of potential beneficial positions was performed by two-step PCR based on the published protocol (Wang and Malcolm 1999). The primers having degenerative nucleotides used for site-saturation mutagenesis are listed in Table S2. The PCR reaction was performed in two steps. In the first step, two separate reactions in separate tubes were performed; one tube containing the forward primer and the another one containing the reverse primer. In the second step, both tubes were mixed and amplification was continued.

The PCR mixture (50 μL) of the first step contained 50 ng backbone plasmid, 1X Q5 standard reaction buffer, 0.2 mM dNTP mix, 200 pM of either forward or reverse primer, and 1 U Q5 DNA polymerase. The PCR was performed as follows: 98 °C for 2 min (1 cycle); 98 °C for 20 s, Tm for 20 s, 72 °C for 8 min (for 3 cycles), and 72 °C for 10 min (1 cycle). After mixing two tubes, another PCR reaction was performed as follows: 98 °C for 2 min (1 cycle); 98 °C for 20 s, primer melting temperature (Tm) for 20 s, 72 °C for 8 min (for 15 cycles), and 72 °C for 10 min (1 cycle). The PCR products were checked on the agarose gel, and subsequently were subjected to DpnI digestion (20 U, 37 °C, overnight). DNA purification of the PCR products was performed using PCR purification kit. Purified PCR products were transformed directly into chemically competent *E. coli* BL21-Gold (DE3) for expression and screening.

### Recombination of compatible substitutions experimentally

The recombination library of the HlyB was generated using an overlap extension PCR method based on the published protocol (Hilgarth and Lanigan 2020). Two oligonucleotides of the *hly*B gene with 6 and 7 substitutions were synthesized by GenScript. Both synthesized oligonucleotides, the *hlyB* wildtype gene, plus the plasmids with improved positions were used as templates for the overlap extension PCR. Subsequently, the HlyB overlap extension library was cloned into the pK184 plasmid using Gibson assembly method and transformed into *E. coli* BL21-Gold (DE3) chemical competent cells.

### Cultivation and expression of libraries in 96-well plates

For each library, single active clones were picked and placed in 150 µL LB_kan_ medium in 96-well plates (PS-F-bottom, Sarstedt, Germany) along with four wildtype clones and four empty vector clones as positive and negative controls, respectively. Active clones were identified by generating halo on the tributyrin-agar plates. The cultures were incubated overnight (24 h, 37 °C, 900 rpm, and 80% humidity) in a 96-well MTP shaker (SI505 MTP shaker incubator, Avantor). Then, 50 μL of glycerol 50% (v/v) were added to each wells and the plates, named as master plates, were stored at − 80 °C for further experiments. For expression, each master plate was sub-cultured into a new 96-well MTP plate containing 150 µL LB_kan_ medium and cultivated (24 h, 37 °C, 900 rpm, and 80% humidity). The cultures were used to inoculate new 96-well MTP plates containing 150 µL LB_kan_ medium and cultivated for 3 to 4 h (37 °C, 900 rpm, and 80% humidity). Subsequently, main cultures were induced with 1 mM of IPTG and 5 mM CaCl_2_. The cultures were further cultivated for 16 h (37 °C, 900 rpm, and 80% humidity) in 96-well MTP shaker incubator. Afterwards, the MTPs were centrifuged (4 °C, 20 min, 2900 g) and supernatants were harvested and used for the further investigation.

### Colorimetric screening system

The screening of the libraries with the *para*-nitrophenyl butyrate (*p*NPB) assay was performed as previously reported in 96-well MTPs (Pourhassan et al. 2022a). Screening of the recombinant library via lipase colorimetric assay proved to be a challenging task as the amount of lipase in the supernatant of clones was high and reached the saturation level of the assay. To solve this issue, the supernatant was further diluted in the assay reaction buffer (20-fold dilution) and the incubation time before inducing the cultures was reduced to 3 h.

### Computational method to analyze stability

The structure of HlyB was modeled based on the structure of PCAT1 (PDB: 4RY2) (Lin et al. 2015) with TopModel tool (Mulnaes et al. 2020). The homology model of HlyB was used for the computational analysis. For visualization, the HlyB /HlyD structure (PDB: 7SGR) driven from Cryo-EM from Zhao et al. work was used (Zhao et al. 2022).

The ΔΔG_fold_ (ΔΔG_fold_ = ΔΔG_fold, Sub_ − ΔΔG_fold, WT_) values of individual substitutions were computed using FoldX method version 3b5.1 (Guerois et al. 2002), in YASARA structure version as described previously (Cui et al. 2020; Van Durme et al. 2011). Internal-atomic distance between 12 beneficial positions of the HlyB wildtype measured by YASARA (Table S3). Default FoldX parameters were used (temperature 298 K; ionic strength 0.05 m; pH 7) to generate the substitutions. Mutate residues commands of FoldX were used to compute the ΔΔG_fold_ values of substitutions (Table S4). PyMOL molecular graphics system, version 1.8.6.0 enhanced for Mac OS X (DeLano 2002) was used for visualization.

## Results

Overall, two KnowVolution campaigns were employed on the HlyA T1SS of *E. coli* inner membrane complex, which resulted in an increased secretion. First, we describe the results of the four phases of the KnowVolution campaign on HlyB. Encouraged by these results, we also conducted a KnowVolution campaign on HlyD to further improve the secretion efficiency.

### KnowVolution of HlyB toward increasing secretion efficiency

**Fig. 1:**
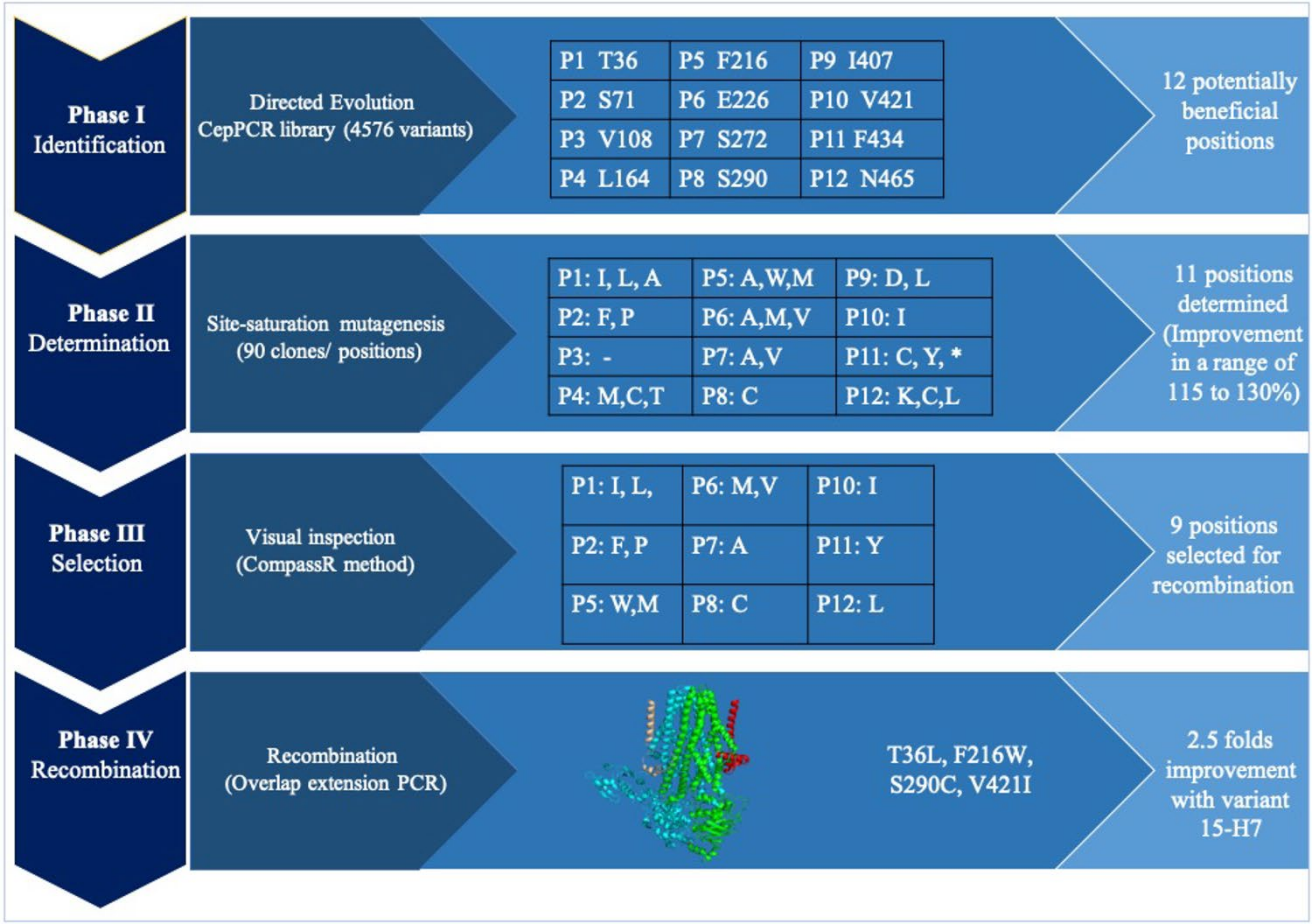
An overview of the HlyB KnowVolution campaign aimed at increasing the secretion efficiency of the HlyA T1SS. In phase I, the hlyB gene underwent random mutagenesis, resulting in the identification of 12 potential beneficial positions. In phase II, all 12 positions were subjected to site-saturation mutagenesis. In phase III, the computer-assisted recombination (CompassR) strategy was used to select substitutions for recombination based on the HlyB homology model. Finally, in phase IV, recombination was performed resulting in a variant harboring four substitutions (T36L, F216W, S290C, V421I). This variant showed a 2.5-fold improvement in secretion efficiency compared to the wildtype

To identify amino acid positions of the HlyB transporter that increase the secretion efficiency of HlyA T1SS, a KnowVolution campaign, in 4 phases was conducted (Fig. 1).

#### Phase I (identification)

In the first phase of KnowVolution campaign on HlyB, beneficial positions of the *hlyB* gene were identified through a random mutagenesis approach known as casting error-prone PCR (cepPCR) according to the published protocol (Yang et al. 2017). In cepPCR approach, the gene of interest is divided into smaller fragments, and subsequently, fragments were subjected to epPCR individually. To generate a library on HlyB using cepPCR approach, the gene of *hlyB* was divided into six fragments (F_1B_: 275 bp, F_2B_: 251 bp, F_3B_: 283 bp, F_4B_: 256 bp, F_5B_: 279 bp, F_6B_: 291 bp). The region of *hlyB* gene encoding the NBD was excluded. Excluding the region of the *hlyB* gene that encodes the NBD was expected to reduce the number of inactive clones in the resulting library, as this region contains highly conserved motifs that are essential for transporter function.

Random mutagenesis libraries of those six fragments were generated using a low-fidelity DNA polymerase in buffer conditions with different MnCl_2_ concentrations (ranging from 0.1 to 0.9 mM). The mutation frequency was adjusted by altering the MnCl_2_ concentration to obtain libraries with an inactive ratio of 40–50%. The inactive ratio of libraries was determined via prescreening clones on tributyrin agar plates, as active clones were able to generate halos because of the secreted lipase-HlyA1, which was used as model substrate. The mutation load of libraries was in the range of 3.6 to 5.2 mutations per fragment (on average almost 15 mutations per kbp).

In the first phase, ~ 4576 clones were screened in a 96-well MTP format using the *para*-nitrophenyl butyrate (*p*NPB) assay to identify *E. coli* clones that secrete the lipase with higher titers. The promising variants showing higher secretion level in compared to wildtype variant were rescreened four times via *p*NPB assay to eliminate the false positive clones. In this phase, the selected promising variants showed increased secretion efficiency in a range of 1.2- to 1.6-fold compared to the parental type (wild type).

Despite generating and screening of a larger library for the fifth fragment, we did not identify any beneficial position. Upon aligning the sequence of the promising variants, it was observed that twelve positions were repeatedly mutated, which were considered as “potential beneficial positions.” These positions were T36, S71, V108, L164, F216, E226, S272, S290, I407, V421, F434, and N465.

#### Phase II (determination)

To obtain the full diversity of those 12 identified positions, individual site-saturation mutagenesis were performed on each position to identify variants with increased secretion levels. A total of 96 variants were screened for each position to ensure that all possible amino acids were represented. From each individual library, clones with higher secretion level in compared to wildtype variant were isolated. To be sure that isolated variant are not false positive, they rescreened four time via the *p*NPB assay. After the rescreening, those confirmed promising variants were sent for sequencing. Table 1 summarizes the substitutions that exhibited higher secretion efficiency compared to the wildtype.

**Table 1:** Sequence analysis of saturated positions in HlyB variants

Positions	Substitutions with improved secretion of targeted lipases	Compatible substitutions suggested by CompassR
Position 1: T36	Ile^*^, Leu, Ala	Ile and Leu
Position 2: S71	Phe^*^, Pro	Pro and Phe
Position 3: V108	**–**	–
Position 4: L164	Met, Cys, Thr^*^	–
Position 5: F216	Ala, Trp, Met^*^	Met and Trp
Position 6: E226	Ala, Met, Val^*^	Met and Val
Position 7: S272	Ala^*^, Val	Ala
Position 8: S290	Cys	Cys
Position 9: I407	Asp, Leu^*^	–
Position 10: V421	Ile	Ile
Position 11: F434	Cys, Tyr, a silent mutation^*^	Tyr
Position 12: N465	Lys^*^, Cys, Leu	Leu

Amino acids highlighted in bold showed higher secretion efficiency compared to other substitutions

#### Phase III (selection)

**Fig. 2:**
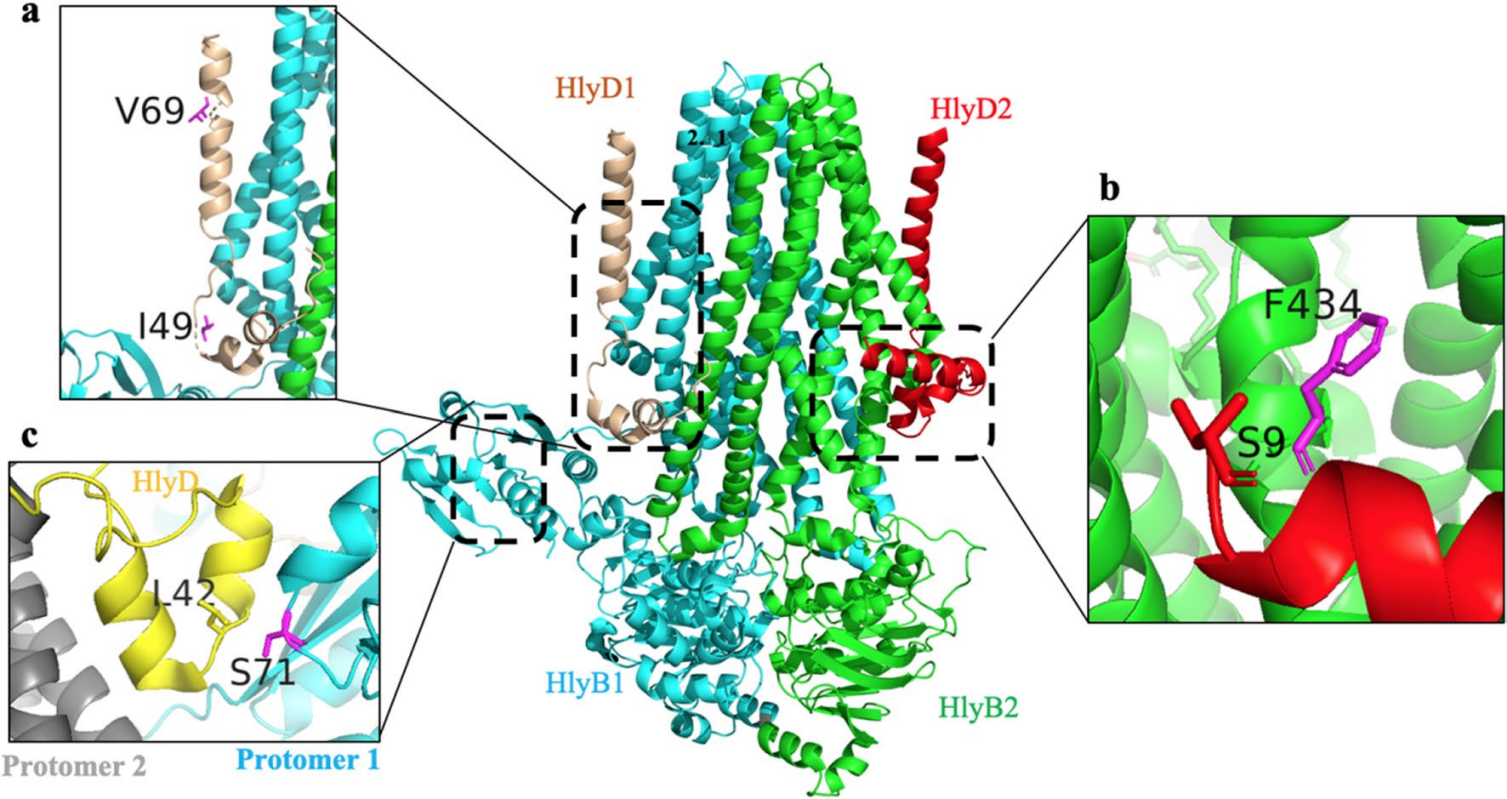
An illustration of the potential beneficial positions in the three-dimensional structure of HlyB wildtype (PDB: 7SGR). HlyB dimer is in cyan and green. **a** I49 of HlyD is among the residues involved in the interaction with the positively charged region of the CLD. **b** F434 of HlyB is located in close proximity of the amphipathic helix of HlyD. The amphipathic helix is oriented parallel to the membrane. **c** Residue S71 is located in the protomer-protomer interface. The figure was generated using the PyMol software (www.pymol.org)

**Fig. 3:**
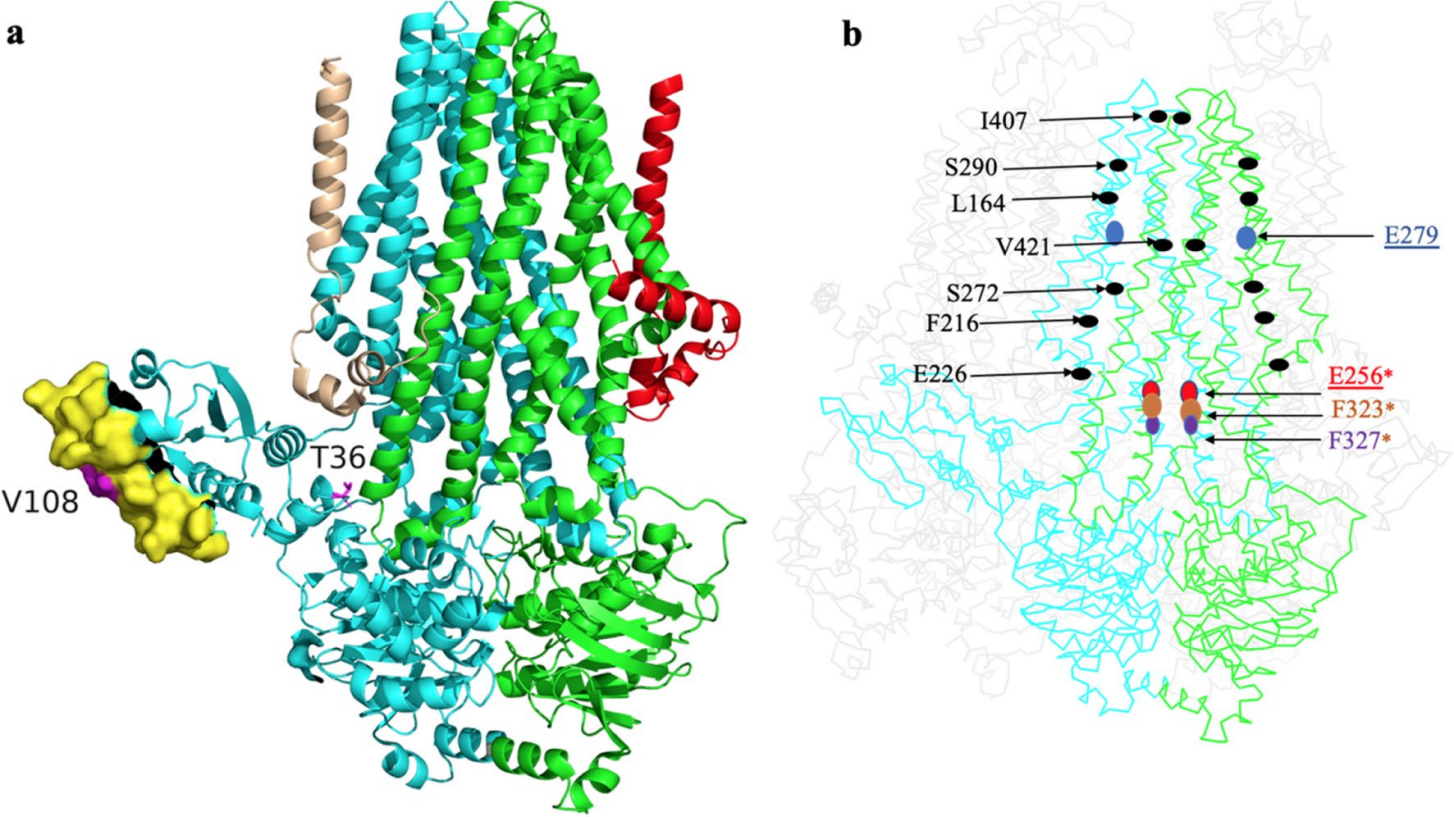
An illustration of the potential beneficial positions in the three-dimensional structure of the HlyB wildtype (PDB: 7SGR). **a** Carton representation of HlyB dimer (cyan and green) associated with two HlyD molecules (light brown and red), with the yellow surface mapping the HlyA-interaction region on the CLD (Lecher et al. 2012). The magenta color highlights residue 108, which is located within this interaction region. **b** An illustration of the identified beneficial positions determined in the current study as black dots. The positions suggested by Holland et al. involved in oligomer formation are underlined (Holland et al. 2016), and the positions at the opening of HlyB suggested by Zhao et al. (2022) are highlighted by an asterisk. The figure was generated using PyMol software (www.pymol.org)

No three-dimensional structure of HlyB was available during the experimental phase of the study. Therefore, we created a homology model based on the crystal structure of the ABC transporter PCAT1 (Lin et al. 2015) to perform the calculations. However, a single particle cryo-EM structure of HlyB/HlyD was recently published (Zhao et al. 2022). This structure (PDB: 7SGR) used for visualization of the identified beneficial positions (Figs. 2 and 3).

To investigate whether the identified beneficial positions exhibit cooperative effects, we explored the recombination of these residues. However, recombining multiple beneficial positions is a major challenge in protein engineering, as it often leads to variants with reduced or even no activity after recombining three or four substitutions (Liebeton et al. 2000). To address this challenge, we employed a computer-assisted recombination (CompassR) strategy, which has been previously validated for improving the performance of the BSLA lipase (Cui et al. 2020). The CompassR strategy relies on the calculation of the relative free energy of folding (ΔΔG_fold_) of different substitutions and enables the in silico selection of substitutions that can be combined for improved protein function. Table 2 summarizes the calculated ΔΔG_fold_ values for HlyB substitutions. Based on the CompassR selection criteria (ΔΔG_fold_ < 0.36), we identified nine beneficial substitutions (Table 1) that were recombined in phase IV.

**Table 2:**
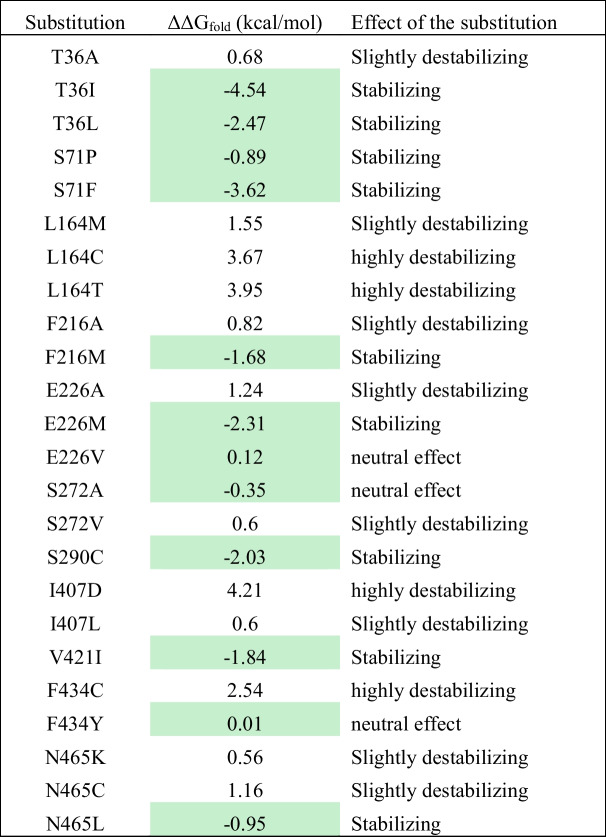
The ΔΔG_fold_ of substitutions and their effect on HlyB. The selected substitutions based on the CompassR criteria (ΔΔG_fold_ < 0.36) are highlighted in green. The larger the ΔΔG_fold_ negative values, the higher the stability

#### Phase IV (recombination)

**Fig. 4:**
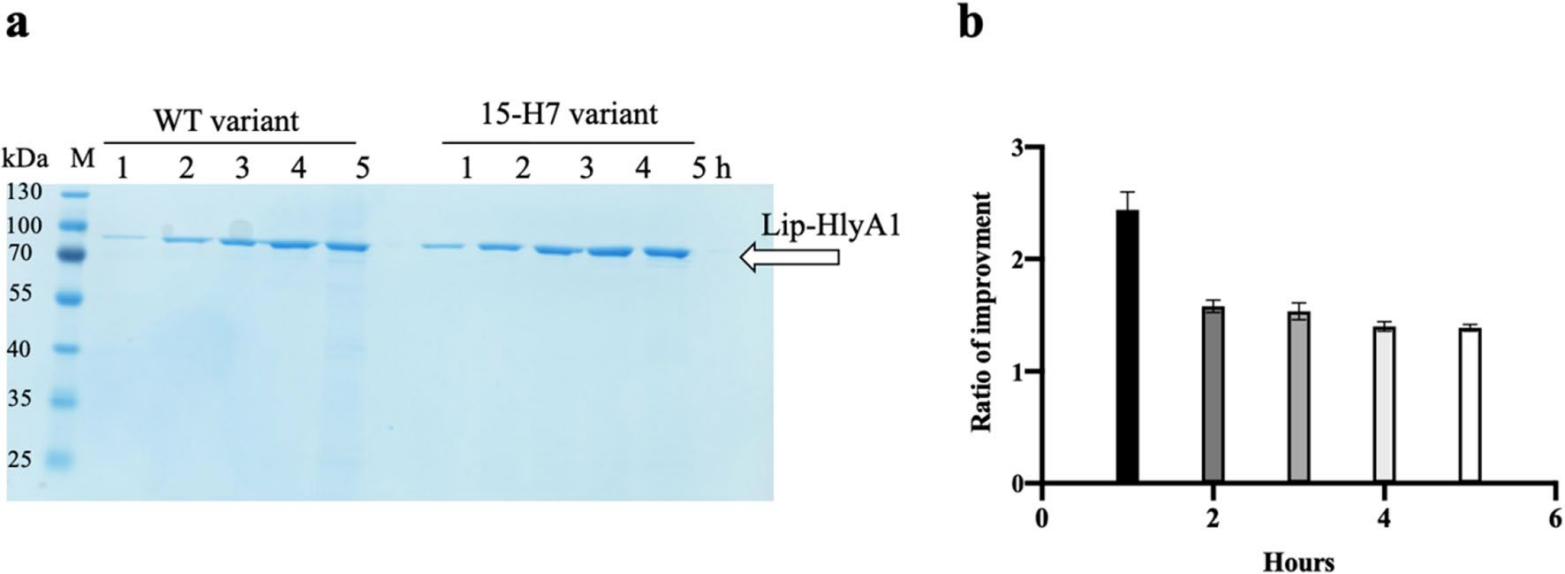
Secretion of lipase-HlyA1 through the HlyA T1SS. **a** SDS-PAGE of the unconcentrated supernatant of wildtype and the 15-H7 variant secreting lipase-HlyA1. **b** Semi-quantification of SDS-PAGE gel via the ImageJ software (Abràmoff et al. 2004). Ratio of the improvements of 15-H7 variant compared to the wildtype. One-way ANOVA (*p* < 0.05) indicated a significance differences between the time points. The error bars are based on three independent replicates with the error bars reporting S.D. The molecular weight of the marker proteins (M) is given on the left (kDa). xh, x hours after induction

Recombination between selected substitutions from phase III was accomplished through an overlap extension PCR according to a published protocol (Hilgarth and Lanigan 2020). The resulting library was estimated to have 1500 different combinations. Variants showing higher secretion levels of lipase were isolated and rescreened four times. The screening of the recombinant library yielded a variant, termed as 15-H7, with an improvement of around 2.5-folds in the secretion of lipase-HlyA1 (Fig. 4a). This variant contained 4 substitutions: T36L, F216W, S290C, and V421I.

### Comparing the secretion level of HlyB wildtype and 15-H7 variant

A test secretion experiment was conducted in 100-mL Erlenmeyer flasks to compare the secretion levels of the 15-H7 variant (the best variant obtained from the HlyB KnowVolution campaign) and the wildtype. The unconcentrated supernatant of both cultures was analyzed by SDS-PAGE (Fig. 4). Prior to loading on the SDS-PAGE, the samples were normalized based on OD_600_ of the cultures. A semi-quantification of the SDS-PAGE gel using the ImageJ software (Collins 2007) confirmed approximately a 2.5-fold increase in the secretion efficiency of the 15-H7 variant of compared to the wildtype at 1 h after induction. However, the improvement decreased to 1.5-fold after the second hour due to aggregate formation, as evident from the formation of white foam in the supernatant of the 15-H7 culture (Fig. 4b). The semi-quantified SDS-PAGE signals were statistically investigated via analysis of variance (ANOVA) and it indicated a significant differences between the different time points.

**Fig. 5:**
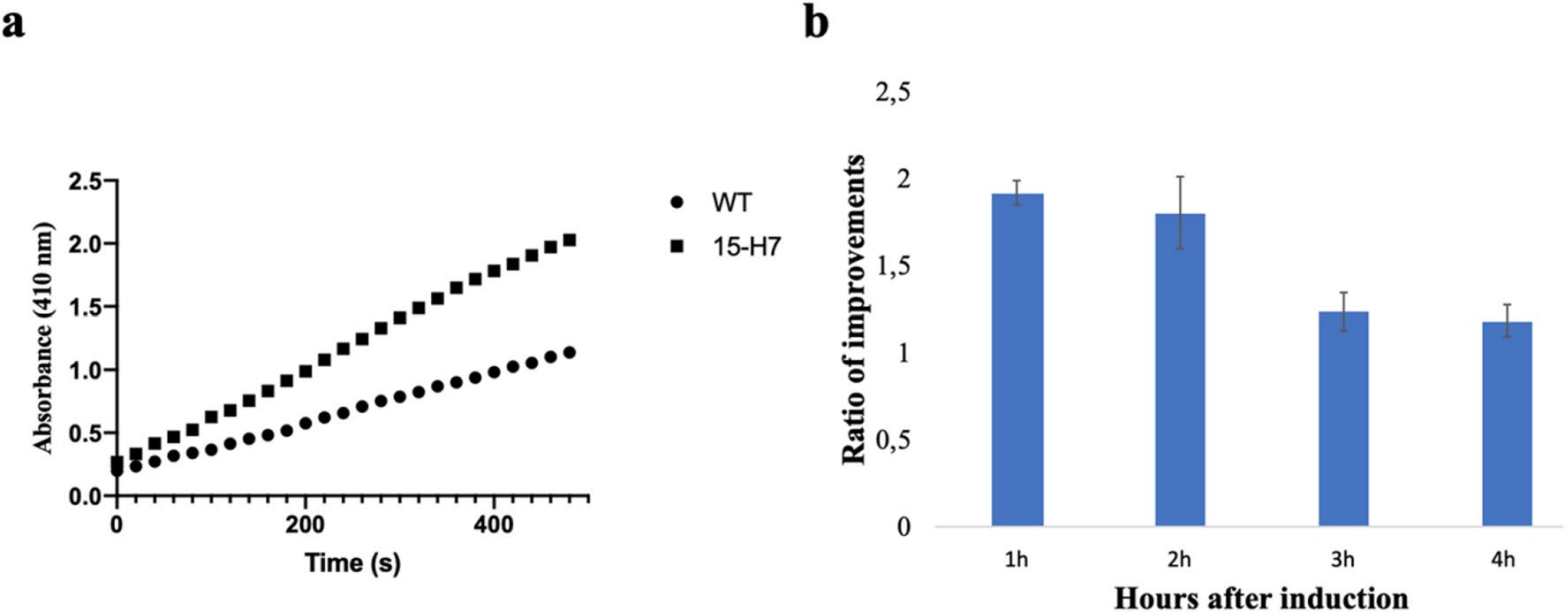
Enhanced secretion of lipaase-HlyA1 by the 15-H7 variant. **a** Activity of the lipase-HlyA1 in the supernatant of the culture dedicated to either the “15-H7” or wildtype HlyB using the *p*NPB assay. **b** Ratio of improved lipase activity of the wildtype and the improved variants using the *p*NPB assay. The error bars are based on three independent replicates with the error bars reporting S.D

In addition, the supernatant samples collected every hour after induction were used to perform the *p*NPB assay to determine lipase activity. The absorbance intensities of samples were measured at 410 nm. The *p*NPB assay results confirmed the SDS-PAGE analysis and showed that the secretion level of 15-H7 was higher than the wildtype (Fig. 5a, b). The improvement in secretion was higher in the early hours after induction, with the amount of soluble enzyme in the 15-H7 culture being 2.5-fold higher than in the wildtype during the first hour after induction. However, the amount of secreted Lipase-HlyA1 in the 15-H7 culture reached a concentration that promoted aggregation, visible as white particles.

**Fig. 6:**
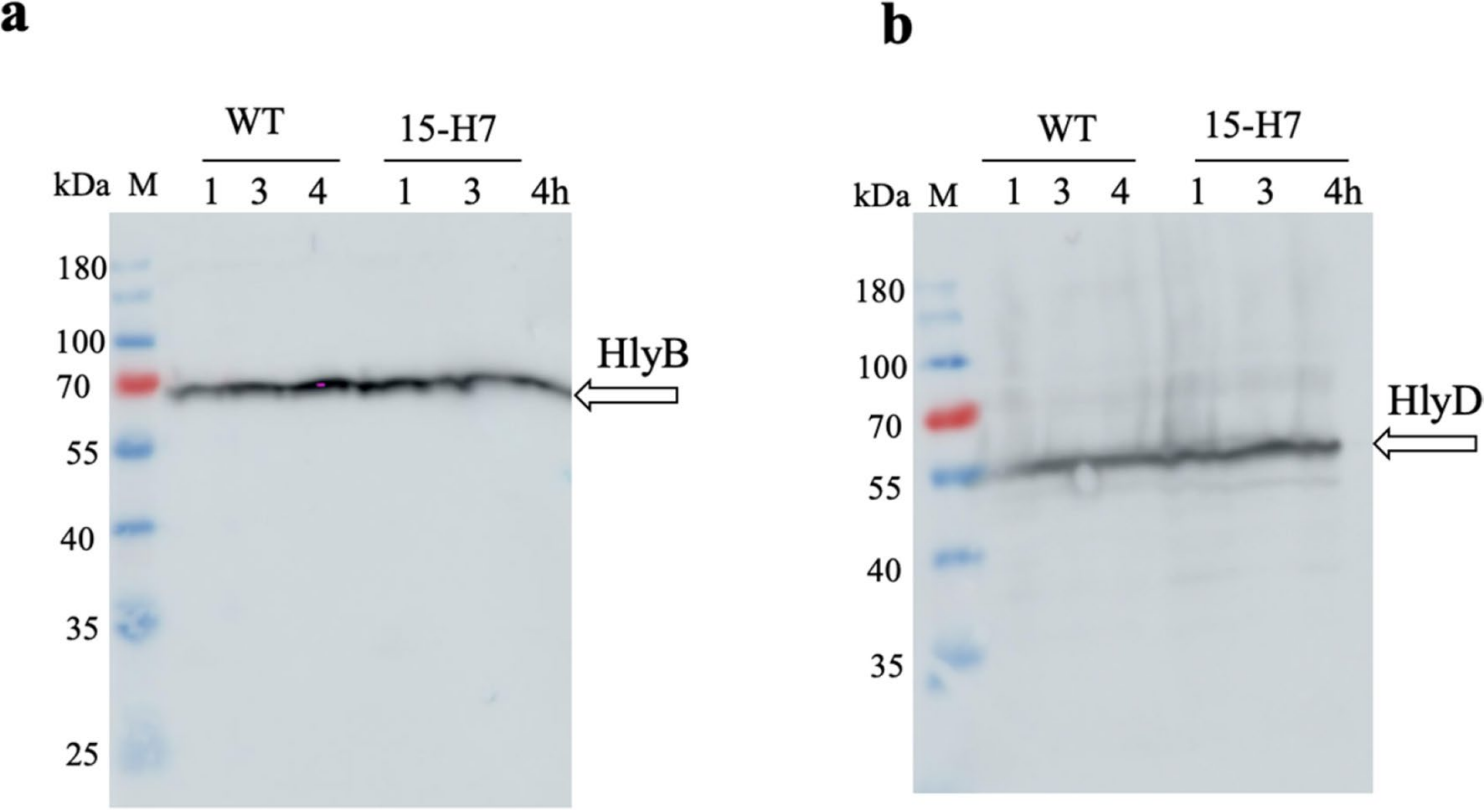
Western blot analysis of *E. coli* cells secreting lipaase-HlyA1 through the HlyA T1SS. **a** Western blot of HlyB and **b** western blot of HlyD using polyclonal antibodies against HlyB and HlyD, respectively. The blots indicate that both membrane proteins were expressed for both variants at comparable levels. The molecular weight (kDa) of the marker proteins (M) is given on the left; xh: cell pellets of culture, where x denotes the number of hours after induction

The level of expression of HlyB (Fig. 6a) and HlyD (Fig. 6b) was analyzed by western blotting for *E. coli* cells having either the wildtype or the 15-H7 variants. This analysis performed in three biological replicates and confirmed that HlyB and HlyD were expressed at comparable levels in both variants. Thus, the improved secretion efficiency of the 15-H7 variant cannot be attributed to a higher expression of the HlyA secretion system apparatus.

To determine the amount of secreted lipase-HlyA1 in the 15-H7 culture, a serial dilution of purified HlyA with known concentration was prepared and a calibration curve was generated. The concentration of secreted lipase-HlyA1 in the supernatant was quantified to approximately 400 mg/L. The amount of lipase-HlyA1 (wet weight) collected in the white particles was estimated to be 5 mg from 25 mL culture (200 mg/L).

### Improved secretion of cutinase-HlyA1 through HlyA T1SS

We were eager to see if the 15-H7 HlyB variant could promote the secretion of a different target protein rather than lipase. To do this, a fusion gene encoding the cutinase protein from *Fusarium solani* f.sp. *pisi* was cloned instead of the lipase gene in the plasmid harboring the 15-H7 HlyB variant. The test expression was conducted for wildtype and 15-H7 variant, and the supernatant of the cultures was analyzed via western blotting. This results showed that the secretion of cutinase-HlyA1 in the presence of the 15-H7 HlyB variant was 2.5-fold higher than the wildtype one. This observation demonstrated that the novel HlyB is capable of enhancing the secretion of another target protein, cutinase-HlyA1 (Figure S1).

### Application of KnowVolution to HlyD for increased secretion efficiency

Following the success in improving HlyB secretion efficiency, we applied the KnowVolution strategy to HlyD in an attempt to further enhance the secretion efficiency of the HlyA T1SS system. The KnowVolution strategy for directed evolution of HlyD was the same as already explained for HlyB.

In the identification step (phase I), we identified three potential beneficial positions located in the cytoplasmic domain of HlyD: M6, I49, V69. However, despite screening site-saturation libraries for these positions, no promising variant was found. We attempted to regenerate the mutation libraries on fragments 1 and 2 to cover more beneficial variants, but still no positions were determined.

## Discussion

Secretion, as a strategy of recombinant protein production, could offer several advantages such as increased stability and solubility of the target protein, as well as a significant reduction in the production costs due to simplified downstream processes (Burdette et al. 2018; Kleiner-Grote et al. 2018).

Note almost one-third of all FDA-approved recombinant proteins are produced by *E. coli* (Puetz and Wurm 2019; Sharma and Chaudhuri 2017), but to our knowledge, there is no example of usage of *E. coli* as a secretion host in industrial scales. Despite the benefits of using *E. coli* in large-scale protein production, the low secretion levels have posed a challenge. To address this issue, researchers have attempted to engineer different secretion systems of *E. coli*, but with little success so far (Burdette et al. 2018; Pourhassan et al. 2021).

T1SS have a relatively simple architecture among other secretion systems of Gram-negative bacteria (Costa et al. 2015), making this class of secretion systems attractive targets for engineering efforts. The best achieved secretion titer for T1SS in different Gram-negative bacteria has been reported for metalloprotease secreted by TliDEF, a T1SS from *Pseudomonas fluorescens*, with a yield of 789 mg/mL (Burdette et al. 2018; Pourhassan et al. 2021).

Previously, we reported on a successful improvement of the secretion efficiency of the HlyA T1SS through a KnowVolution approach on the HlyA enhancer fragment, as well as including a terminator region. Our previous work resulted in a secretion level of approximately 180 mg/L for soluble lipase-HlyA1. Additionally, the white particles in a small culture volume of only 25 mL had a high protein content estimated to be above 1.5 mg of lipase-HlyA1 (Pourhassan et al. 2022a).

So far two case studies attempted to apply directed evolution for engineering of the inner membrane proteins of the Hly secretion system, but both reported studies were restricted to traditional protein evolution methods, epPCR (Eom et al. 2005; Sugamata and Shiba 2005). In the current study, we applied for the first time a combination of directed evolution and computational assisted strategy, CompassR, to explore the protein sequence of the inner membrane proteins of the Hly secretion system more effectively. It has already reported that applying cepPCR for random library generation increases the chance of identifying the beneficial positions of a gene of interest (Yang et al. 2017).

In the current work, we applied KnowVolution strategy on the inner membrane complex of the system to further optimize secretion of target proteins, resulting in significant improvements in secretion efficiency. Our T1SS system achieved now more than 400 mg/L soluble lipase plus around 5 mg in form of aggregated enzyme in a culture volume of only 25 mL through a KnowVolution strategy.

We acknowledge that the optimized HlyA T1SS in the current study is still away from being a potential platform in industrial scales for different substrates, but the obtained improvement indeed indicates that putting more efforts on the HlyA system is worth as there might be still rooms for improvement in efficiency of the HlyA system. It might also be beneficial using modern machine learning technologies to explore the sequence space of different system components either individually or in the context of each other. Latter one might be achieved by the provided practical supports from a deeper understanding of the HlyA T1SS secretion mechanism in the future.

In contrast to previous KnowVolution campaigns that used epPCR or SeSAM (Ji et al. 2020) for diversity generation, we employed a cepPCR method (Yang et al. 2017) that enables us to adjust mutation frequencies to small peptide stretches. This approach led to the identification of several beneficial amino acid positions within the cepPCR library for HlyB, with 12 positions distributed in the CLD and TMD. Furthermore, the CompassR strategy (Cui et al. 2020) was performed in the phase III of the HlyB KnowVolution campaign. The best variant after all four phases contained four mutations: T36L/F216W/S290C/V421I.

During the KnowVolution campaign, three positions (T36, S71, and V108) were identified located in the CLD, which is the domain that interacts with HlyA before secretion (Lecher et al. 2012). Recently, Zhao et al. showed that an interaction between HlyD and the CLD is essential for secretion. They also defined the main interface between the three protomers, which is a projection between the cytosolic domain of HlyD, residues 35–50, with a positively charged surface of the CLD. Interestingly, one of the positions we identified as potentially beneficial for HlyD, I49, is located within this region of HlyD. In addition, another identified possible beneficial of HlyD, V69, is involved in making contact with TM1/2 of HlyB. However, site saturation of these positions did not result in any improved variants.

One potential position of HlyB, S71, is located in the inter-protomer interface in close proximity to the residues involved in the HlyD/CLD interaction. In the work by Zhao et al., the S71C mutation was tested and led to the conclusion that oligomerization between HlyB and HlyD occurs in the native environment, which is a prerequisite for secretion (Zhao et al. 2022).

Previous reports have indicated that the central part of the CLD interaction site is composed of hydrophobic residues, and upon interaction with substrate, chemical shifts were observed in the first α-helix and β-sheets number 3 to 5 of the CLD (Lecher et al. 2012). Notably, the identified potential beneficial position V108 is located within the β-sheet 5 of the CLD.

Experiments conducted by Holland laboratory using cross-linking reported that two HlyB mutants, E256K and S279L, were unable to form oligomers with HlyD (Holland et al. 2016). In the work by Zhao et al., residues R256, F323, and A327 were identified as being located at the lateral opening of the protomer involved in HlyA translocation. Furthermore, they identified several positions of HlyB that were in close proximity to the substrate eGFP-HlyA1, including positions 256, 263, 319, 323, 327, and 422 (Zhao et al. 2022).

Our suggested possible beneficial positions of HlyB, including F216, E226, S272, and S290, appear to be in the vicinity of the substrate interaction region of the TMD of HlyB. Especially, V421, which was identified as a beneficial positions in the current study, is located within the translocation pathway (Zhao et al. 2022).

Interestingly, the potential beneficial position I407 is located in the periplasmic loop P3 which is between TMHs 5 and 6. This loop comprising 8 residues in HlyB is conserved in a wide variety of ABC transporters. A site-saturation mutagenesis study on the residues of this loop demonstrated that at least four residues of this loop are essential for secretion, as for instance, I401T and D404G mutants showed only 20% of hemolytic activity and a double mutant, S402P and D404K, showed no hemolytic activity at all (Holland et al. 2016).

Structural analysis shows that our beneficial position, F434, is located exactly at a location in which the amphipathic helix of HlyD (residues 9–24) contacts the elbow helix of HlyB (Zhao et al. 2022).

The KnowVolution campaign in HlyD did not identify any single residue for improving the secretion efficiency of HlyA T1SS. This is consistent with two previous directed evolution studies on HlyA T1SS, which also did not identify any improved mutants harboring HlyD mutations (Low et al. 2010; Sugamata and Shiba 2005). One possible explanation for this is that only 60 residues of HlyD are located in the cytosol, limiting its direct contact with the substrate to these residues. The rest of the protein is either located in the membrane (20 residues) or in the periplasmic space (approximately 400 residues). Another possibility is that HlyD is already optimized by evolution and is in its best functional state.

The applied evolutionary approach of this study can be generally applied to other section systems to improve the secretion performance of bacterial secretion systems. An improved secretion system opens up an avenue for the usage of Gram-negative bacteria such as *E. coli* as a secreting host for industrial protein production.

## Supplementary Information

Below is the link to the electronic supplementary material.

Supplementary file1 (PDF 493 KB)

## Data Availability

The datasets generated during and/or analyzed during the current study are available from the corresponding author on reasonable request.
