# Supplementary material for: A step forward to the optimized HlyA type 1 secretion system through directed evolution
Source: Appl Microbiol Biotechnol. 2023 Jul 5;107(16):5131–43. doi: 10.1007/s00253-023-12653-7 (PMC10386944; doi:10.1007/s00253-023-12653-7)
Supplement: Supplementary file 1 — Supplementary file1 (PDF 493 KB) [file 253_2023_12653_MOESM1_ESM.pdf]

Supplementary materials

Article in Applied Microbiology and Biotechnology

A step forward to the optimized HlyA type 1 secretion system through directed evolution

Zohreh N. Pourhassan,[a] Haiyang Cui,[b,c,f] Neele Muckhoff,[a] Mehdi D. Davari,[d] Sander H. J. Smits,[a] Ulrich Schwaneberg,[b,c] and Lutz Schmitt*[a]

[a]
Institute of Biochemistry,
Heinrich Heine University,
Universitätsstr. 1, 40225 Düsseldorf (Germany)
E-mail of corresponding author: lutz.schmitt@hhu.de

[b]
Institute of Biotechnology,
RWTH Aachen University
Worringerweg 3, 52074, Aachen (Germany)

[c]
DWI-Leibniz Institute for Interactive Materials
Forckenbeckstraße 50, 52056 Aachen (Germany)

[d]
Department of Bioorganic Chemistry
Leibniz Institute of Plant Biochemistry
Weinberg 3, 06120 Halle (Germany)

[f]
Present address: Carl R. Woese Institute for Genomic Biology
University of Illinois at Urbana-Champaign
1206 West Gregory Drive, Urbana, Il 61801 (USA)

Table S1 Primers used for epPCR amplification of fragments

Fragments	Forward primer (5' to 3')	Reverse primer (5' to 3')
F_{1B} (275 bp)	GCCACATAAGATTGCTATTTTTTG	CCATCCTCTCTCCAGACTAATG
F_{2B} (251 bp)	TCTGGAGAGAGGATGGACGTC	AACAAGGGTTTCAATAAATATTCTC
F_{3B} (283 bp)	GAATATTTATTGAAACCCTTGTTGTG	CGACTCTCAAAATAAGAGATCGG
F_{4B} (256 bp)	CTCTTATTTTGAGAGTCGTCGTG	CCGTGAAAACTTATCATCAAGGC
F_{5B} (279 bp)	TGATGATAAGTTTTCACGGAATGCG	GCAATTAACTGACCAATCGATAAATCC
F_{6B} (291 bp)	CTGGTTATTTCCGGGGATTTATCG	CCCCTGCTTAATACTGAGATTG
F_{1D} (279 bp)	CAGTTACAGTCAGACTAACAG	TTCCACCTGACCTAAAAC
F_{2D} (260 bp)	GGTTTCTGGTTATTGCTG	AATTCAATTGACCTGCTC
F_{3D} (289 bp)	CGGTATCAAATTCTGAGCAG	CCTGTTTATGCAATAAACTCC
F_{4D} (290 bp)	GGTTGAAAAAAGCCGTCTG	AAGCCTGTTGACGCTCTTC
F_{5D} (280 bp)	GCATTGAGTTATTAACTCTGGAG	CCCACCAGATAACCATATC
F_{6D} (287 bp)	CCTTTCCTTACACCCGATATG	CCGGCCGCTCTAGATTAC

Table S2 Primers used for site-saturation at potential beneficial positions

Beneficial positions	Forward primer	Reverse primer
SSM of potential beneficial positions of HlyB		
Ter 36	CATAGATTTGACNNKGACGG	CCGTCMNNGTCAAATCTATG
Ser 71	GATTAAACTTTATTNNKCTGCCCG	CGGGCAGMNNAATAAAGTTTAATC
Val 108	GAAATCCCCGTNNKCTCGAACAG	CTGTTCGAGMNNACGGGGATTTC
Leu 164	GTCTGTTTTTNNKCAATTATTTG	CAAATAATTGMNNAAAAACAGAC
Phe 216	GAACTTACATTNNKGCACATAGTACAAG	CTTGTACTATGTGCMNNAATGTAAGTTC
Glu 226	GATTGATGTTNNKTTGGGTG	CACCCAAMNNAACATCAATC
Ser 272	GACAGGCATTAACANNKGTTCTG	CAGAACMNNTGTTAATGCCTGTC
Ser 290	GTGGTATTACNNKCCAAAG	CTTTGGMNNGTAATACCAC
Ile 407	GATTTATCGNNKGGTCAGTTAATTG	CAATTAACTGACCMNNCGATAAATC
Val 421	GCTGGTCAGATTNNKGCACCG	CGGTGCMNNAATCTGACCAGC
Phe 434	GCACAAATCTGGCAGGATNNKCAGCAG	CTGCTGMNNATCCTGCCAGATTTGTGC
Asn 465	GGAAATTNNKGGTGATATCACTTTTCG	CGAAAAGTGATATCACCMNNAATTTCC
SSM of potential beneficial positions of HlyD		
Met 6	CATGGTTANNKGGGTTCAG	CTGAACCCMNNTAACCATG
Ile 49	GGAATTANNKGAAACGCCG	CGGCGTTTCMNNTAATTCC
Val 69	GTTTCTGNNKATTGCTGTC	GACAGCAATMNNCAGAAAC

Table S3 Internal-atomic distance between 12 beneficial positions of the HlyB wildtype measured by YASARA. The distance values were reported with Å unit.

	T36	S71	V108	L164	F216	E226	S272	S290	I407	V421	F434	N465
**T36**	0	26.3	27.6	53.1	40.9	30.1	40.2	63.0	70.9	49.3	29.2	37.5
**S71**		0	18.2	62.1	54.5	48.1	46.2	62.1	72.7	52.8	36.3	59.4
**V108**			0	52.3	46.2	44.1	38.5	50.6	59.4	40.5	27.7	65.0
**L164**				0	14.5	29.4	16.5	24.3	26.7	16.4	26.2	72.6
**F216**					0	15.5	15.0	36.4	39.4	22.6	19.1	58.5
**E226**						0	22.8	48.3	54.1	31.0	20.8	43.2
**S272**							0	26.4	34.6	15.4	12.3	64.1
**S290**								0	13.1	15.5	34.2	90.3
**I407**									0	21.7	42.3	96.9
**V421**										0	20.6	76.5
**F434**											0	57.7
**N465**												0

Table S4 $\Delta\Delta G_{fold}$ (kcal/mol) values of amino acid substitutions on the twelve beneficial positions of the HlyB calculated using FoldX method.

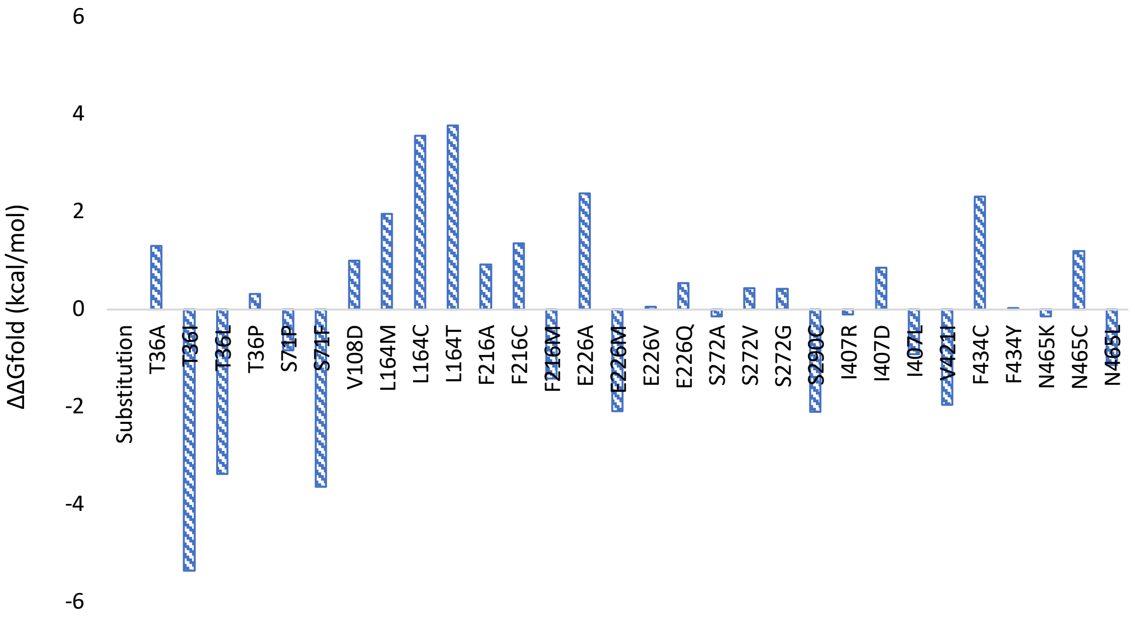

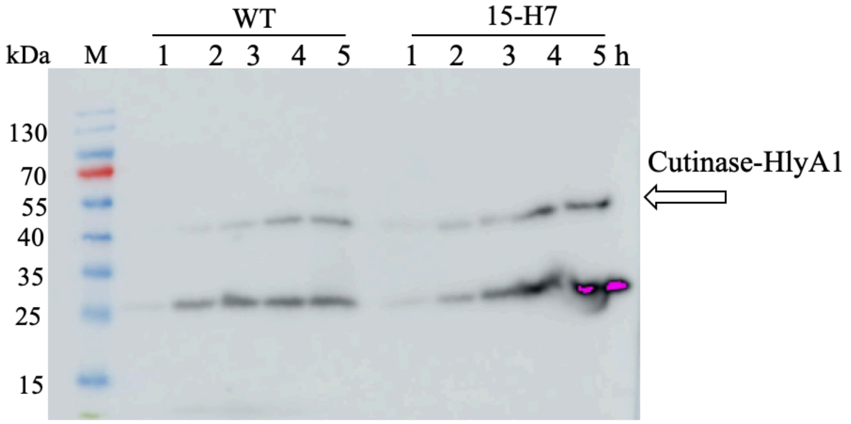

Fig S1 Improved secretion of cutinase-HlyA1 by the 15-H7 variant. a) Western blot analysis of supernatant fractions from the 15-H7 variant the wildtype cultures. The molecular weight (kDa) of the marker proteins (M) is given on the left; xh: cell pellets of culture, where x denotes the number of hours after induction. This experiment performed in triplicate.

HlyD KnowVolution

Phase I (Identification): In the first phase, beneficial positions of HlyD were identified through the cepPCR as already explained above for the HlyB. Based on the cepPCR approcah, the gene of *hly*D was divided into six fragments (F_{1D}: 279 bp, F_{2D}: 260 bp, F_{3D}: 289 bp, F_{4D}: 290 bp, F_{5D}:280 bp, F_{6D}: 287 bp). All steps of library generation and screening were performed as already explained for the HlyB protein.

For each fragment, a library with a size of 786 was generated. Subsequently, the libraries were screened in a 96-well MTP format using the *p*NPB screening to identify clones with higher secreted lipase-HlyA1. None of the fragments, except of the first fragment, showed promising variants. The promising variants of the first fragment were sent for sequencing. Alignment of the sequence of this promising variants identified three potential beneficial positions, listed as: M6, I49, V69.

Phase II (determination): To obtain full diversity on the identified beneficial positions, the two beneficial positions were subjected to individual site-saturation mutagenesis. In this step no promising variant were found after screening of the site-saturation libraries for none of the identified positions. To be sure about the outcome of the KnowVolution of HlyD, a new libraries were generated on the fragments 1-2. But still no positions were found.
